# Redox Imbalance and Stroke

**DOI:** 10.1155/2016/3065263

**Published:** 2016-07-19

**Authors:** Ruidong Ye, Ming Shi, Qian Liu, Jieli Chen

**Affiliations:** ^1^Department of Neurology, Jinling Hospital, Medical School of Nanjing University, Nanjing 210002, China; ^2^Department of Neurology, Xijing Hospital, The Fourth Military Medicine University, Xi'an 710032, China; ^3^School of Dentistry, Cardiff Institute of Tissue Engineering and Repair, Cardiff University, Heath Park, Cardiff CF14 4XY, UK; ^4^Department of Neurology, Henry Ford Hospital, Detroit, MI 48202, USA

Redox status is correlated with mitochondrial function, lipid raft turnover, and cellular cross talk in the neurovascular coupling. Redox imbalance is a hallmark event in the pathophysiology and prognosis of stroke. Excessive reactive oxygen species (ROS) can cause oxidative damage to various biological macromolecules including DNA, lipids, and proteins, thereby altering several signaling pathways that ultimately promote cellular damage and death. It is critical to address the relationship between redox status and stroke and to translate the accumulated mechanistic knowledge from animal models to clinical settings.

This special issue is devoted to highlighting novel findings on the role of redox imbalance in stroke and possibilities of the antioxidant supplementation for stroke treatment.

In this issue there are two investigations focused on the contribution of systemic inflammatory status to pathophysiology and prognosis of ischemic stroke patients. In a cross-sectional study, lower serum level of caveolin-1, which has been reported to maintain blood-brain barrier integrity and counteract oxidative stress, was found to be significantly related to cerebral microbleeds in patients with acute ischemic stroke. However, no relationship was observed between caveolin-1 and the presence of silent lacunar infarcts and white matter hyperintensities, two other types of cerebral small vessel disease. Though the relatively small sample size (~156 patients) may cripple the power of the evaluation, this study strongly supports caveolin-1 as a biomarker for predicting cerebral microbleeds in acute ischemic stroke. Maintaining caveolin-1 level appeared to be a potential strategy in treating cerebral small vessel diseases.

X. Zhang et al. carried out a prospective study on the predictive effects of the presence of metabolic syndrome (MetS) on early neurological deterioration following acute ischemic stroke. Interestingly, systemic markers of inflammation, fibrinogen and high-sensitivity C-reactive protein, were not the mediators of the relation between MetS and early neurological deterioration. The authors addressed that local oxidative or inflammatory response markers may be more appropriate when involved in studies of stroke and other potential correlated factors.

Two investigations focused on how comorbidity affects stroke outcome and therapeutic approaches. Stroke patients with diabetes mellitus have worsened mortality and neurological recovery. Bradykinin receptors have been demonstrated to play important roles in cerebral ischemia/reperfusion injury in the context of diabetes. R. Shi et al. carried out a study to elucidate the differential roles of bradykinin B1 receptor and B2 receptor underlying the effect of tissue kallikrein on the stroke outcome in diabetic rats. The results revealed that tissue kallikrein protects against inflammatory reactions by suppression of microglial activation and neutrophil infiltration through bradykinin B2 receptor, rather than B1 receptor. The bradykinin B2 receptor-dependent protection requires the activation of ERK/CREB/Bcl-2 signaling pathway to counteract apoptosis and neuroinflammation. Another potent antioxidant agent, minocycline, was investigated by E. A. Fontes-Júnior et al. in cortical ischemic injury combined with chronic alcohol intoxication. The results showed that chronic alcohol intoxication increased nitrite and lipid peroxidation levels and neuronal loss caused by ischemia. Minocycline was effective in preventing histological and neurological damage caused by stroke alone. Nevertheless, the protection of minocycline was abolished when stroke was preceded by chronic alcohol intoxication. This study suggests that ischemic stroke combined with chronic alcohol intoxication is more severe and may require different therapeutic strategies.

J. Qu et al. provided a comprehensive review to summarize the role of ROS in the intracerebral hemorrhage. ROS is mainly released from neurons exposed to hemoglobin metabolic products after intracerebral hemorrhage, which is strikingly different from the pathophysiology in ischemic stroke. This ROS is harmful to the central nervous system through cell death and structural damage, especially the disruption of the blood-brain barrier. The review continues to discuss how endogenous antioxidant system interacts with the oxidant agents and why previous antioxidant therapy failed to confer practical neuroprotection in clinical trials. The authors suggest more attention be paid to the local and systemic influences of ROS in intracerebral hemorrhage and the longitudinal profile of ROS burst after intracerebral hemorrhage.

In another review article, J. Bu et al. discussed the effects of a natural product, omega-3 polyunsaturated fatty acids (n-3 PUFAs), the major component of fish oil, on the redox imbalance after stroke. Due to their antioxidation, anti-inflammation, and interaction with pathways such as Nrf2/HO-1 signaling, n-3 PUFAs are neuroprotective in reducing infarction volume and improving neurogenesis and revascularization. The authors also expressed that further studies were warranted to establish the specific treatment strategy of n-3 PUFAs in stroke.

In conclusion, the goal of this special issue is to drive a better understanding of how redox imbalance modulates pathophysiology and prognosis of stroke and how this can be harnessed as a therapeutic target in the stroke treatment. The original and review articles in this issue contributed by the experts in the fields of redox and stroke will nurture and expand knowledge of the mechanisms behind oxidative damage.

*Ruidong Ye* *Ruidong Ye* *Ming Shi* *Ming Shi* *Qian Liu* *Qian Liu* *Jieli Chen* *Jieli Chen*

